# Characterization of chloroplast genome of the marine diatom *Chaetoceros gracilis*

**DOI:** 10.1080/23802359.2021.1987171

**Published:** 2021-10-07

**Authors:** Yajun Li, Xiuxia Zhang, Ru Zeng, Xiaodong Deng

**Affiliations:** Hainan Provincial Key Laboratory for Functional Components Research and Utilization of Marine Bio-resources, Institute of Tropical Bioscience and Biotechnology, Hainan Academy of Tropical Agricultural Resource, Chinese Academy of Tropical Agricultural Sciences, Haikou, P. R. China

**Keywords:** *Chaetoceros gracilis*, chloroplast genome, phylogenetic analysis, diatom

## Abstract

In the present study, the chloroplast genome of *Chaetoceros gracilis* was sequenced using the PacBio sequencing platform and phylogenetic analysis was conducted using 38 other complete chloroplast genomes of the Bacillariophyta. The chloroplast genome of *C. gracilis* was 116,421 bp in length with the typical quadripartite structure, including a large single copy (LSC) region of 61,904 bp, a small single copy (SSC) region of 39,367 bp, and a pair of inverted repeats (IR) regions of 7575 bp. The overall GC content of *C. gracilis* chloroplast genome was 30.79%. This genome encoded 131 genes incuding 93 protein-coding genes, 30 transfer RNA (tRNA) genes and 8 ribosomal RNA (rRNA) genes. Phylogenetic results exhibited that three Chaetoceros species were clustered together. *Chaetoceros gracilis* was closely related with *Chaetoceros muelleri*, and then formed a clade with *Chaetoceros simplex* with 100% bootstrap value This study will facilitate species identification and study of evolutionary in the family Chaetoceroceae.

*Chaetoceros gracilis* Pantocsek 1892 is a marine centric diatom belonging to the genus Chaetoceros Ehrenberg in Chaetoceroceae (Chaetoceratales, Bacillariophyta), which is widely used as food for bivalves or shellfish due to its high eicosapentaenoic acid (EPA) and fucoxanthin contents (Brown and Blackburn 2013; Tachihana et al. 2020; Hassan et al. 2021). In addition, as a photosynthetic eukaryote, it also contributes to the research of photosynthesis. For instance, a stable oxygen-evolving Photosystem II complex from *C. gracilis* was successfully isolated and purified (Nagao et al. 2010), and recently, Nagao et al. (2020) investigated the effects of CO_2_ concentration and temperature on the photosynthetic performance in *C. gracilis*. Moreover, the structures of photosystem I-fucoxanthin chlorophyll a/c proteins (PSI-FCPI) and PSII-FCPI supercomplex from *C. gracilis* were solved by single-particle cryo–electron microscopy (Pi et al. 2019; Xu et al. 2020). However, little information was available about the chloroplast genome of *C. gracilis,* and little was known about the plastid genome evolution within order-level clades In this study, we obtained the complete chloroplast genome of *C. gracilis* using Pacbio and Illumina sequencing technologies, the sequence and genome annotation are available in GenBank under accession number MZ352931.

*C. gracilis* strain CCMA-291 was provided by the Center for Collections of Marine Algae, Xiamen University, China (N24.61°, E118.32°), the strain was isolated from the entrance of the Yangtze River into the East China Sea. The Chloroplast DNA was isolated with Plant Chloroplast DNA column extraction kit (BioRab, Beijing) according to the instructions of the manufacturer, and sequenced by combining Illumina Hiseq4000 and PacBio sequencing platform at Nextomics Biosciences Co. Ltd (Wuhan, China). The specimen was deposited at the herbarium of institution of Tropical Bioscience and Biotechnology, Chinese Academy of Tropical Agricultural Sciences under the voucher number XXJMZ5. The detailed sequencing methods were as described as Li and Deng (2021). In total, 4444.3 Mb of PacBio subreads were generated, and 8440.8 Mb Illumina clean reads were yielded after filtering out containing N, low quality reads and adapter related sequences by using Trimmomatic 0.39 (Bolger et al. 2014). The software NOVOPlasty v2.7.2 (Dierckxsens et al. 2017) was employed for De novo assembly of the chloroplast genome with *Chaetoceros simplex* (GenBank: NC_025310.1) as the reference. GapCloser V1.12 software (Luo et al. 2012) was used to perform vulnerability completion and base correction. The genome was annotated on the online tool GeSeq (Tillich et al. 2017).

The total chloroplast genome of *C. gracilis* was 116,421 bp in length, with a GC content of 30.79%. The genome harbored a typical quadripartite structure with a large single copy region (LSC, 61,904 bp) and a small single copy region (SSC, 39,367 bp) separated by two copies of an inverted repeat (IR, 7,575 bp). A total 131 genes were predicted in the whole chloroplast genome, containing 30 tRNAs, 8rRNAs, and 93 protein-coding genes, none of which contain introns. Moreover, four rRNAs (*rns, rnl, rrn23* and *rrn5*), three tRNAs (*trnp-UGG, trnl-GAU*, *trnA-UGC*) and two protein- coding genes (*psbY* and *ycl89*) were located in the IR regions. Ribosomal protein 32 (*rpL32*) was located in the border of IRA and SSC, and ycf45 was located in the border of IRA and LSC.

We compared the chloroplast genomes of *Chaetoceros gracilis*, *Chaetoceros muelleri* and *Chaetoceros simplex* using Mauve alignment. The result revealed that these genomes exhibited a collinear relationship, as only one syntenic block from each strain was present (Figure S1) (Darling et al., 2004). And then, the junction sites were visualized using IRscope web tool with default parameters (https://irscope.shinyapps.io/irapp/; Amiryousefi et al. 2018). The expansion and contraction of IR regions could result in the chloroplast genome size slight variation. The rpl20 genes among the three Chaetoceros species were all found in LSC region and faraway from the border of LSC/IRb with 46 bp in *C. gracilis*, 39 bp in *C. muellerii* and 57 bp in *C. simplex*. A different IRb/SSC gene arrangement pattern was observed in *C. simplex* where the duplicate psbY gene was situated at the IRb region. The rpl32 gene was located in SSC/IRa region but extended with 64 and 58 bp away from the junction in *C. gracilis* and *C. muellerii*, respectively, whereas it was completely located in SSC region by a 40 bp spacer from the junction in *C. simplex* (Figure S2).

**Figure 1.**
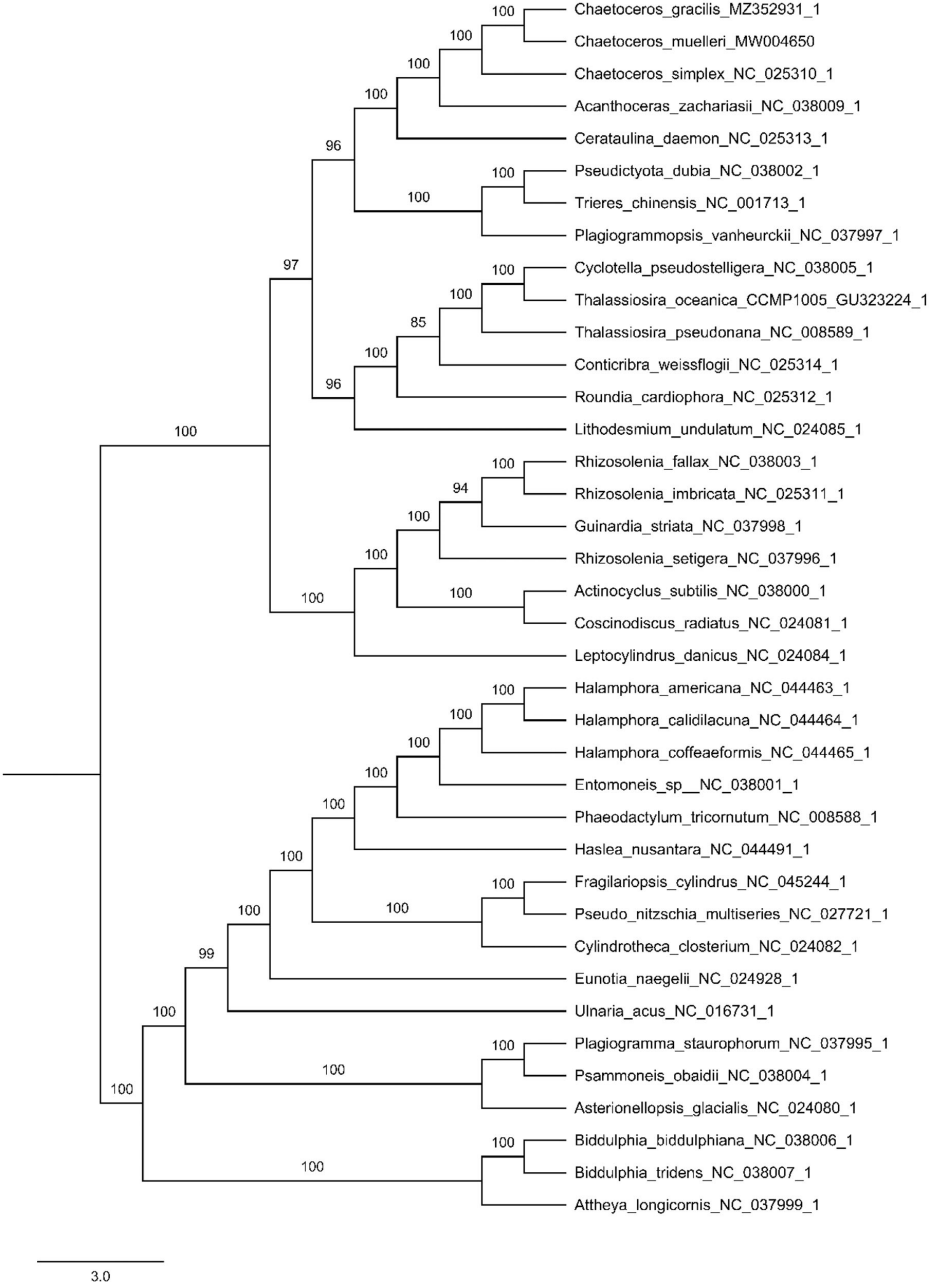
Phylogenetic relationships of 38 species based on concatenated coding sequences of 115 chloroplast coding genes. The phylogenetic analysis was performed by using the software PhyloSuite. The sequences were aligned by MAFFT v7.037 and concatenated, and then the data was partitioned using PartitionFinder2 with AICc model selection under GTR, GTR + G and GTR + I + G + X models. The IQ-tree was used to infer the maximum likelihood (ML) tree with 5000 ultrafast bootstraps under Partition Mode.

A phylogenetic analysis was performed using maximum likelihood (ML) in PhyloSuite using the concatenated coding sequences of 115 chloroplast coding genes for 38 species of Bacillariophyta (Zhang et al. 2020). Supports for nodes were calculated via 5000 ultrafast bootstrap replicates. The results showed that *C. gracilis* was sister to *C. muellerii*, forming a clade with *C. simplex* (Figure 1). The complete chloroplast genome sequence of *C. gracilis* will provide useful information for understanding its phylogenetic resolution and molecular identification.

## Data Availability

The genome sequence data that support the findings of this study are openly available in GenBank of NCBI at (https://www.ncbi.nlm.nih.gov/) under the accession no. MZ352931. The associated BioProject, SRA, and Bio-Sample numbers are PRJNA739799, SRS9249448, and SAMN19803003, respectively.
